# Supplementation of Broiler Chicken Feed Mixtures with Micronised Oilseeds and the Effects on Nutrient Contents and Mineral Profiles of Meat and Some Organs, Carcass Composition Parameters, and Health Status

**DOI:** 10.3390/ani12131623

**Published:** 2022-06-24

**Authors:** Malwina Zając, Bożena Kiczorowska, Wioletta Samolińska, Renata Klebaniuk, Dariusz Andrejko, Piotr Kiczorowski, Szymon Milewski, Anna Winiarska-Mieczan

**Affiliations:** 1 Institute of Animal Nutrition and Bromatology, University of Life Sciences, Akademicka Street 13, 20-950 Lublin, Poland; zajac.malwina1@gmail.com (M.Z.); wioletta.samolinska@up.lublin.pl (W.S.); renata.klebaniuk@up.lublin.pl (R.K.); xszymon47@gmail.com (S.M.); anna.mieczan@up.lublin.pl (A.W.-M.); 2 Department of Biological Bases of Food and Feed Technologies, University of Life Sciences, Głęboka Street 28, 20-612 Lublin, Poland; dariusz.andrejko@up.lublin.pl (D.A.); piotr.kiczorowski@up.lublin.pl (P.K.)

**Keywords:** oilseeds, micronisation, chicken meat, minerals, nutrients

## Abstract

**Simple Summary:**

In the present study, the inclusion of micronised camelina, flax, and sunflower seeds in the diets of broiler chickens improved the slaughter parameters, for example, the share of commercial cuts in the carcass (breast, thigh, and drumstick muscle), and reduced the content of abdominal fat. The addition of oilseeds in the diets also modified the calorific value of the breast, thigh, and drumstick muscles as well as some organs by reducing the content of ether extract. In addition, the flax seeds increased the content of Ca, Cu, and Fe in the muscles and organs. At the same time, there were no abnormalities in the health status of the birds.

**Abstract:**

In this study, we included 15% doses of infrared-irradiated camelina, flax, and sunflower seeds in the diets of broiler chickens (grower and finisher) and focused on assessing the effects on the production traits, selected slaughter analysis parameters, nutrients, and minerals in breast and drumstick meat and some organs, i.e., liver, proventriculus, and heart. In total, 200 one-day-old broiler chickens were assigned randomly to four treatments with five replicate cages of 10 broiler chickens per cage (five females and five males). The experiment lasted 6 weeks. In the group of broiler chickens in which diets were supplemented with micronised camelina and flax seeds, there was an increase in (*p* < 0.05) breast, thigh, and drumstick weight and a decrease (*p* < 0.05) in the abdominal fat of the carcasses. The oilseed treatments reduced the ether extract content and the calorific value in the breast and drumstick muscles and organs. The flax seeds contributed to an increase in the contents of Ca (breast muscle and liver), Cu (breast muscle and proventriculus), and Fe (drumstick muscle and heart). Likewise, some blood parameters were influenced by supplementation with infrared-irradiated camelina and flax seeds, for example, there was a decrease in the haemoglobin level and the mean corpuscular haemoglobin concentration (*p* < 0.05). The oilseed treatments also modified the contents of Fe and Ca in the blood plasma of broiler chickens (*p* < 0.05). It may be concluded that infrared-irradiated camelina, flax, and sunflower seeds can be regarded as good diet components exerting positive effects on the dietary value of poultry meat and organs used in dietetics.

## 1. Introduction

Growing consumer nutritional awareness has been accompanied by a demand for the availability of food products on the food market with high, stable, and scientifically verified nutritional and dietary value, for example, meat and offal, i.e., internal organs used in cuisines. The high nutritional value of these food products is determined by their chemical compositions of basic nutrients and their mineral profiles. The nutritional value of meat in poultry production can be modulated through controlled nutritional programs [1,2,3]. One method for dietary modification of nutrient content, especially in terms of regulating the calorific value of poultry muscles and organs, is based on the inclusion of high-fat seeds to feed mixtures [4,5]. Consumers expect that regional feed materials or those common to traditional local cuisines are used in the production of foods of animal origin [6]. Hence, seeds of local oilseed plants, for example, camelina, flax, or sunflower, seem to be valuable [7,8,9].

One of the effects of poultry nutrition with camelina, flax, or sunflower seeds is the increased dietary value of poultry meat, i.e., lower fat content and an improved ratio of n-6/n-3 fatty acids [10,11,12]. Researchers have also reported that supplementation of poultry diets with oilseeds has a positive effect on rearing performance and slaughter parameters [13,14].

The nutritional value of camelina, flax, and sunflower seeds in poultry production may be largely limited by their content of substances with antinutritional properties, for example, linatin, cyanogenic glycosides, phytins, trypsin inhibitors, lignins, and saponins [15,16]. They may, to some extent, limit the growth and production potential of broiler chickens [11]. Methods employed for fodder processing can help to eliminate the negative impact of the antimetabolites contained in oilseeds. Thermal methods, such as micronisation, are a particularly effective solution to this problem [17].

There is still little information in the literature about the effect of the addition of micronised oilseeds to chicken feed mixtures on the content of macronutrients and micronutrients in poultry meat and internal organs. Therefore, the aim of this study was to analyse the impact of the use of micronised high-fat seeds (camelina, flax, and sunflower) in feed mixtures for broiler chickens on the nutritional and dietary quality of meat and offal in terms of basic nutrient contents and mineral profiles. Additionally, the impact of the supplementation on basic slaughter and blood parameters reflecting the health status of the chickens was assessed.

## 2. Materials and Methods

### 2.1. Oilseeds, Experimental Birds, and Management

The experimental mixtures were supplemented with micronised oilseeds. Camelina (*Camelina sativa* L. Crantz) cv. Luna, flax (*Linum* L.) cv. Opal, and dehulled sunflower (*Helianthus* L.) cv. Lech were used as the main source of energy. The seeds for the micronisation process were purchased as certified plant material from Centrala Nasienna (Lublin, Poland). All oilseeds were micronised twice at a temperature of 180 °C for 60 s, heated with infrared rays in a radiation generator ESC-1 with a power output of 400 W (Elcer, Rzeszotary, Poland). A Raynger ST60 infrared thermometer (Raytek, Inc., Santa Cruz, CA, USA) was used to measure the temperature. The chemical composition of the micronised seeds was determined and is presented in Table 1. The analyses were carried out using 250 g samples in 3 repetitions.

This study was approved by the Second Local Ethics Committee of the University of Life Sciences in Lublin under no. 35/2015. The procedures and protocol followed the official animal welfare guidelines and regulations. The vitality rate of the experimental birds was 100%, and no abnormal signs were observed during the experiment. In total, 200 one-day-old broiler chickens were used in the experiment (Ross 308, Cracow, Malopolskie Province, Poland). All birds were randomly assigned to 4 dietary treatments with 5 replicate cages per treatment (5 females/5 males per cage). The experiment was carried out for 6 weeks according to the guidelines on rearing broilers, following the recommendations on the lighting program, with optimal levels of temperature and humidity, and veterinary care [18].

The basal feed mixtures were optimised with the use of cereal meal middlings (wheat and corn) and post-extraction soybean meal, as recommended by [19] (Table 2).

The broiler chickens were fed a standard broiler chicken nutrition programme (3 types of diets). All the diets were iso-energetic and iso-nitrogenous. The mixtures for the youngest broiler chickens (starter) were crumbled, while older birds received feed pellets (grower and finisher). From rearing Day 22, 15% of the micronised oilseeds were included as an experimental factor into the broiler diets in accordance with the methodological assumptions of the experiment.

### 2.2. Sample Collection and Chemical Analyses

Twenty birds (2 females and 2 males per pen) were selected from every treatment for slaughter by decapitation. The body weight of the chickens selected for slaughter was close to the average value in the group. A simplified dissecting analysis was carried out for sampling the meat, proventriculus, liver, and heart [20].

Fat was extracted from the seeds using the diethyl ether solvent with the Soxhlet extraction method [21]. Gas chromatography was used for determining the fatty acid composition [22,23]. The details of the fatty acids analyses were as described by Kiczorowska et al. [9].

The analytical procedures for determining the contents of tocopherols [24], xanthophylls, and total phenolics in oilseeds were similar to those presented in an earlier publication [9].

The amino acid contents in the diets were determined using the AOAC method [22] and the Arnoldi procedure [25]. Cysteine and methionine were determined after oxidative hydrolysis [25].

The mineral contents (Ca, Mg, P, Cu, Fe, and Zn) in the biological samples (meat, organs, diets) were measured (3 replicates/59AA-6300, Shimadzu Corp., Tokyo, Japan). Calcium was determined at λ = 422.7 nm, magnesium at λ = 285.2 nm, copper at λ = 324.8 nm, iron at λ = 248.3 nm, and zinc at λ = 213.9 nm [1,2,26]. The phosphorus level was determined colorimetrically [27]. The Standard Reference Material NCS ZC73016 was used in the chemical analyses of minerals in the chicken meat.

The content of basic nutrients in the infrared-irradiated oilseeds and diets was determined [22]. The energy value of the oilseeds was calculated using specialized formulas for determining metabolisable energy corrected to zero N balance (MEn) [28] and muscle energy using the Atwater system [29].

All the analyses were performed in triplicate and all data were expressed as means.

### 2.3. Analysis of Haematological Parameters and Blood Minerals

Blood was collected from the chickens selected for slaughter (two broiler chickens/cage). Ten hours before blood sampling, the chickens were not given any feed but were provided with continuous access to water. Blood was sampled from the ulnar vein (vena cutanea ulnaris) in the morning before the slaughter. Blood samples for haematological analyses and determination of elements were collected in 2 mL Vacutest tubes with a K_3_EDTA anticoagulant and 6 mL Vacutest tubes containing lithium heparin, respectively, (Vacutest Kima s.r.l. Arzergrande, PD, Italy).

The haematology analysis of blood was performed within three hours after sampling. The red blood cells (RBC) were determined with routine methods [30]. The packed cell volume (PCV) and haemoglobin (HGB) content were determined using a haematological analyser (ABACUS Junior Vet, Diatron, Vienna, Austria). Other parameters of the red blood cell system (the mean cell volume (MCV), mean cell haemoglobin (MCH), and mean corpuscular haemoglobin concentration (MCHC)) were calculated with the use of standard formulas [31].

Plasma was obtained for the analysis of the elements by centrifugation of whole blood at 3000 rpm (603× *g*) for 15 min in a laboratory centrifuge (MPW-350R, MPW Medical Instruments, Warsaw, Poland) at a temperature of 4 °C. Plasma without haemolysis signs was analysed. The content of elements (calcium, magnesium, phosphorus, copper, iron, and zinc) was determined in blood plasma using a biochemical analyser (Metrolab SA, Buenos Aires, Argentine) with the use of reagent kits (BioMaxima, Lublin, Poland and Hydrex Diagnostics, Warsaw, Poland).

### 2.4. Statistical Analysis

The data were analysed with one-way analysis of variance ANOVA (α = 95, *p* < 0.05) and calculation of the mean values for the treatments and the standard error of the mean (Statistica version 13.3, USA). The cage served as the statistical unit. The Shapiro–Wilk and Brown–Forsythe tests were used for testing the normality of data and homogeneity of variances, respectively. Significant differences between the means were determined by Tukey’s honestly significant difference (HSD) post hoc test.

Y_ij_ = μ + a_i_ + e_ij_

where Y_ij_ is the measured variable, μ is an overall mean, a_i_ is treatment, and e_ij_, is the random error.

## 3. Results

### 3.1. Carcass Composition

The addition of 15% micronised oilseeds to the feed mixtures improved the slaughter parameters of the broiler chickens; especially, the abdominal fat content in the carcass was reduced (*p* = 0.016) (Table 3). The content of abdominal fat, analysed muscle, and chosen organs is shown as a percent of final live body weight. In the final rearing period, the average final body weights of the broiler chickens were as follows: Control (2425 g), experimental treatment CAM.IR (651 g), experimental treatment FLA.IR (2631 g), and experimental treatment SUN.IR (2574 g). The values were presented in our earlier publication [32]. The final body weight was influenced by the body weight achieved by the broiler chickens in the subsequent rearing stages: In the grower period, 1709 g in the control and, on average, 1810 g in the experimental treatments; in the experimental (grower and finisher) period, 2067 g in the control and, on average, 2214 g in the experimental treatments. The growth parameters also determined the other production factors, for example, BWG (body weight gain) and FCR (feed conversion ratio). In the grower period, BWG was 1268 g/chicken in the control and was improved in the experimental treatments by approximately 3.4% (CAM.IR), 4.5% (FLA.IR), and 2% (SUN.IR). Similar values of the parameters, i.e., on average, 545 g/chicken per cage, were recorded in all groups in the finisher period [32]. In the fattening period, the FCR index (*p* = 0.019) was also significantly improved from 2.03 in the control to, on average, 1.92 in the experimental treatments [32].

The carcasses of birds receiving mixtures supplemented with the micronised oilseeds were characterised by a higher proportion of breast (*p* = 0.019), thigh (*p* = 0.022), and drumstick (*p* = 0.031) muscles. The carcasses of broiler chickens fed mixtures with the micronised oilseeds, especially sunflower seeds, had higher proventriculus weight (*p* = 0.027) than the birds in the control. No significant changes were observed in the liver and heart weight.

### 3.2. Basic Nutrients and Mineral Elements in Broiler Chicken Muscles and Organs

Supplementing broiler feed mixtures with infrared-irradiated oilseeds contributed to reductions in the content of ether extract by approximately 15% in breast muscles (*p* = 0.031) and approximately 37% in drumstick muscles (*p* = 0.027), as compared with those in the control (Table 4).

The lowest ether extract content was determined in the muscles of broiler chickens that received the micronised sunflower seeds (17.5% and 40% reduction in the breast and drumstick muscles, respectively). The calorific value of the drumstick muscles of the experimental chickens was approximately 17% lower (*p* = 0.018) than that in the group of chickens fed with the standard mixtures. The addition of micronised flax seeds (FLA.IR) to the feed mixtures resulted in the largest reduction (by approximately 19.5%) of the calorific value of the drumstick muscles as compared with that of the control. No significant differences were found in the content of the other nutrients in the analysed poultry muscles.

Similar changes were also observed in the content of basic nutrients in the organs of the experimental broiler chickens (Table 5).

As compared with the control treatment, supplementation of the feed mixtures for the chickens with micronised oilseeds reduced the fat content in all the analysed organs: on average, by 7.4% in the liver (*p* = 0.067), 14.4% in the proventriculus (*p* = 0.041), and 11.8% (*p* = 0.027) in the heart. The lowest level of ether extract was determined in the organs of broiler chickens from the FLA.IR treatment as compared with the control chickens. The reduction in the ether extract content in the analysed organs did not significantly reduce the calorific value of these parts of broiler carcass. In terms of the other basic nutrients, the CAM.IR- and SUN.IR-supplemented broiler chickens had significantly higher protein content in the proventriculus (by approximately 15.2% and 16.8%, respectively) than the control chickens.

The mineral profile in the broiler chickens meat was also analysed (Table 4). The breast muscles of chickens from the CAM.IR and FLA.IR variants exhibited higher (*p* = 0.018) Ca content (by 11.8% and 19.6%, respectively) as compared with that of the control chickens. The addition of micronised flax seeds to the feed mixtures increased the content of Cu by approximately 27% in the breast muscles (*p* = 0.041). As compared with the control group, the content of Fe was higher by 7.8% in the breast muscle (*p* = 0.017) and by 11.5% in the drumstick muscle (*p* = 0.024).

Similar modifications were observed in the mineral profiles of the experimental broiler chickens’ organs (Table 5). In the group of macronutrients, the Ca content in the livers of the CAM.IR, FLA.IR, and SUN.IR broiler chickens increased (*p* = 0.024) by approximately 14.1, 26.3, and 8.5%, respectively, as compared with that in the control group. Other significant changes were observed in the levels of the Cu and Fe micronutrients. The addition of 15% micronised oilseeds to the mixtures increased the Cu content (*p* = 0.015) in the proventriculus of broiler chickens, especially in the CAM.IR and FLA.IR treatments (by 11.3 and 14.5%, respectively) as compared with that in the control group. In turn, the Fe content in the heart of the experimental chickens was, on average, 22.3% higher than in the control group.

### 3.3. Haematological Indices and Blood Minerals in Broiler Chickens

The values of the analysed haematological indices and the level of some elements in the blood plasma are presented in Table 6. They are consistent with the reference range specified for this species [30,33,34].

The results indicate that supplementation of broiler chicken diets with micronised camelina (CAM.IR) and flax (FLA.IR) seeds reduced the haemoglobin level and mean corpuscular haemoglobin concentration in the blood as compared with the control group and the birds that received the micronised sunflower additive (SUN.IR) (*p* = 0.013 and *p* = 0.007, respectively). The CAM.IR, FLA.IR, and SUN.IR treatments did not induce changes in the levels of any other haematological parameters (*p* > 0.05).

The addition of micronised flax and sunflower seeds to the diet increased the plasma iron concentration (*p* < 0.003) as compared with that of the control group (Table 6). The SUN.IR and CAM.IR treatments contributed to reduction in the calcium concentration of the chickens’ blood plasma as compared with that of the control group (= 0.023). Supplementation of the feed mixtures with micronised oilseeds did not affect the level of other elements in the blood plasma (*p* > 0.05).

## 4. Discussion

Oilseeds are regarded mainly as a source of fat, especially given their high content of valuable and health-enhancing polyunsaturated fatty acids (PUFA). They also contain antinutritional substances, which can substantially reduce the feed conversion ratio and nutrient availability. This, in turn, may lead to a decline in the efficiency of animal production. Raw flax and sunflower seeds contain trypsin inhibitors. Camelina seeds are additionally characterised by the presence of glucosinolates [35]. In turn, flax seeds contain linamarin, mucilages, and other cyanogenic glycosides [10]. Young birds are particularly sensitive to these factors, which may slow down the rearing process considerably [6,36]. Thermal processes, including micronisation, may effectively reduce the negative impact of antinutritional substances on the rearing performance, without elimination of the positive impact of the high nutritional value of fat on the dietary quality of meat and internal organs.

A beneficial effect of supplementation of feed mixtures with thermally processed oilseeds, i.e., reduction in the abdominal fat content of broiler carcass, was also reported by Parveen et al. [36]. The authors used thermally processed flax seeds in poultry nutrition and observed reduced crude fat content in breast and thigh muscles. The results are in line with those presented in this study. As explained by Anjum et al. [37], the reduced fat content in muscles and internal organs of broilers fed mixtures with thermally processed flax seeds was associated with the high availability of long-chain fatty acids in the feed. A similar phenomenon was also reported by Liu et al. [38], who conducted an experiment on ducks and reported a significantly higher efficiency of conversion of longer-chain FAs C20:5(n-3) than in the case of C22:6(n-3). Ducks receiving a diet rich in this type of fat were characterised by high accumulation of lipid droplets in the liver. The authors suggested that the diets enriched with various fatty acids had a strong influence on PUFA deposition in tissue lipids. Supplementation with various levels of extruded flax seed was found to contribute to a significant reduction in the fat content of breast and thigh meat. A similar phenomenon, consistent with the present results, was also reported by Pietras and Orczewska [39]. The authors showed that the inclusion of camelina oil to the feed mixture contributed to a reduction in abdominal fat of carcasses. A decrease in the fat content of breast and leg muscles in quails was also reported by Jakubowska et al. [40] in experiments based on supplementation of feed mixtures with 4% and 7% of flax seed. The authors emphasised that the mechanism of the modification of adipose tissue deposition by the presence of PUFA in feed has not been fully elucidated.

PUFAs are also believed to be involved in the induction of mitochondrial uncoupling proteins, which may reduce the dietary energy in animals fed an n-3 PUFA-rich diet. This energy can be dissipated or can be used to increase protein deposition [41,42,43]. The authors reported an increase in the protein content in breast muscles accompanied by reduced levels of fat, which confirmed this thesis. A similar mode of protein and fat deposition was also noted in the present study, especially in the internal organs (proventriculus and heart).

As explained by Parveen et al. [36], the higher liver, heart, and kidney weights in the group of birds supplemented with extruded flax seeds were associated with increased protein synthesis in these tissues. These data correspond with the results presented in this study. As compared with the control group, the proventriculus weight was significantly higher in the experimental variants (CAM.IR, FLA.IR, and SUN.IR), and the liver and heart exhibited a tendency toward an increase in weight.

There is little information in the literature on the use of micronised oilseeds in poultry production. There are, however, reports of the effect of supplementation of poultry diets with thermally processed (e.g., extruded) oilseeds. In a study on broiler chickens, Anjum et al. [37] analysed the effect of supplementation with extruded flax seeds. The authors found that the administration of extruded flax seeds in the chicken diet significantly improved the quality as well as the dietary and functional properties of poultry meat. Similar studies were conducted by Zhaleh et al. [44], who analysed the effect of the addition of 5, 10, and 15% extruded flax seeds to the diet in the last rearing period (finisher). The authors concluded that the 10% dose of the experimental seeds in the broiler diet proved to be beneficial for production efficiency as compared with the birds fed with the standard mixture (control).

There is insufficient information in the literature about the effect of diets containing raw and thermally processed high-fat seeds on the mineral composition in muscles. The higher concentrations of Fe and Cu in the muscles, Cu in the proventriculus, and Fe in the heart in the CAM.IR, FLA.IR, and SUN.IR treatment groups may be associated with the high blood levels of these elements, to some extent reflecting their concentrations in the entire organism. The levels of iron and copper in an organism largely depend on the diet and the degree of gastrointestinal absorption [45]. Equally interesting is the higher level of Ca in the breast muscles and liver of the CAM.IR and FLA.IR broiler chickens as compared with the control. As demonstrated by investigations of broiler chickens conducted by Gümüş et al. [45], an adequate level of Ca in the diet inhibits the intensity of lipid peroxidation in meat with maintenance of adequate dietary nutrient proportions in meat. The authors proposed that the combination of Ca sources with natural antioxidants in the diet could be used to improve carcass characteristics and antioxidant capacity in broiler meat.

In the present study, the content of other minerals in the breast and drumstick muscles and the analysed organs did not differ significantly, irrespective of the energy source in the diet (infrared-irradiated camelina, flax, and sunflower seeds). This issue requires further multidirectional research. Nevertheless, the present results suggest some possibilities of modulation of the level of minerals, especially the haematopoietic micronutrients, in meat and internal organs used by consumers as the dietary source of these elements.

The analysis of selected haematological indices was performed to assess the possible adverse effects of the micronised oilseeds on the health status of the animals. The dietary inclusion of camelina and flax seeds to the diet significantly decreased the haemoglobin level and MCHC (haemoglobin-related index) as compared with those in the control diet and the sunflower seed treatment. Our previous study analysed the addition of raw oilseeds in broiler chicken nutrition also indicated a decrease in the haemoglobin level in supplemented groups [46]. Other studies conducted with the use of two flaxseed varieties in rabbit nutrition have shown a decrease in RBC, HGB, MPV, and PCV in the blood of the experimental animals [47]. The reduction in the level of haemoglobin and MCHC induced by the FLA.IR supplementation may be related to the content of cyanogenic glycosides (linamarin, linustatin, and neolinustatin) and enzymes (*β-bis*-glucosidase, *β*-monoglucosidase, and *α*-hydroxynitrile lyase), which are involved in the hydrolysis of cyanogenic glycosides and the release of hydrocyanic acid [35,48,49]. Hydrogen cyanide and cyanide salts are toxic to an organism, as they block cellular respiration enzymes and inhibit the activity of other enzymes due to their ability to bind with iron, manganese, or copper ions, which are part of the functional groups of many enzymes. Hydrogen cyanide has the ability to bind with blood haemoglobin to form cyanohaemoglobin, which does not dissociate into haemoglobin [50,51]. In turn, camelina seeds contain glucosinolates (glucoarabin, glucocamelinin, and 11-(methylsulfinyl)-undecylglucosinolate), which do not pose a threat to animal health, but their degradation products, for example, isothiocinate, nitrile, and thiocynate, exhibit multidirectional biological activity [35].

The determination of metals and other elements in biological material, for example, in blood plasma, is highly important for assessing not only environmental exposure but also the proper supply and transformation of elements in organisms [52,53]. The FLA.IR treatment was observed to modify the content of Fe in the blood, muscles, and organs. The concentration of iron in blood depends on Fe abundance in the diet and on the absorption of this element in the gastrointestinal tract and the intensity of the decomposition and synthesis of haemoglobin [54]. Probably, the presence of cyanogenic glycosides in flax seeds and their influence on the formation of cyanomethemoglobin mobilises iron stores and increases its blood plasma level. Other anti-nutrients present in oilseeds, for example, phytic acid, tannins, and glucosinolates, may affect the availability and status of these elements in an organism as well [15,16,35,40].

## 5. Conclusions

The 15% addition of infrared-irradiated camelina, flax, and sunflower seeds in grower and finisher diets improved the dietetic value of the meat and some organs and some slaughter parameters in broiler chickens. The micronised camelina and flax seeds in the feed mixture decreased the abdominal fat content in broiler carcasses and increased the weight of breast, thigh, and drumstick muscles and some internal organs. The oilseed treatments reduced the ether extract content and the calorific value in the breast and drumstick muscles and the analysed organs (liver, proventriculus, and heart). The micronised experimental oilseeds added to the feed mixtures contributed to an increase in the contents of Ca, Fe, and Cu in the breast and drumstick muscles and the content of Ca in the liver, Cu in the proventriculus, and Fe in the heart. The effect of the micronised oilseed treatments on some blood parameters did not negatively affect the health status of the animals. There is a need for further investigations to elucidate the mechanisms associated with the ability of oilseeds to potentiate the element retention. To sum up, micronised camelina, flax, and sunflower seeds can be considered to be good diet components that exert positive effects on the dietary value of poultry meat.

## Figures and Tables

**Table 1:** Chemical composition of infrared-irradiated camelina, sunflower (dehulled), and flax seeds.

Compounds	Camelina	Flax	Sunflower	SEM ^2^
Basic nutrients, g/kg dry matter				
Dry matter	949.3	967.5	973.1	5.33
Crude ash	41.5	36.4	35.6	0.07
Crude protein ^1^	209.2	218.3	189.4	3.41
Ether extract	403.8	422.9	531.7	4.52
Crude fibre	83.5	39.1	36.6	0.73
Fatty acids, g/100 g ether extract				
C16	6.51	6.49	9.73	0.06
C18	3.59	5.63	4.55	0.09
C16:1	0.09	0.09	0.08	<0.01
C18:1	15.33	16.82	28.49	0.06
C18:2	16.42	15.29	57.63	0.38
C18:3	31.98	55.47	0.08	0.77
SFA ^3^	13.37	12.48	12.17	0.05
MUFA ^4^	33.87	17.85	28.34	0.31
PUFA ^5^	53.12	70.47	58.64	0.82
Minerals, mg/100 g natural matter				
Ca	368.2	119.2	276.3	8.37
P	738.1	68.3	653.7	21.9
Mg	398.7	44.6	325.1	13.7
Cu	1.16	1.37	0.69	0.03
Fe	14.1	5.41	8.43	0.21
Zn	7.15	5.09	4.74	0.11
Bioactive components, total in fresh matter				
Tocopherols, μg/g	378.9	187.3	142.3	4.18
Xanthophyll, μg/g	37.3	15.3	29.8	0.13
Phenolics, mg/100 g	648.7	76.7	167.8	0.25

Results are the average of three analyses, ^1^ calculated by Kjeldhal nitrogen N × 6.25. ^2^ SEM, standard error of the mean; ^3^ SFA, saturated fatty acid; ^4^ MUFA, monounsaturated fatty acids; ^5^ PUFA, polyunsaturated fatty acids.

**Table 2:** Dietary ingredients and nutrient content in the experimental diets.

Component	Diets ^1^											
	Starter (0 to 21 Days)				Grower (21 to 35 Days)				Finisher (35 to 42 Days)			
	Control	CAM.IR	FLA.IR	SUN.IR	Control	CAM.IR	FLA.IR	SUN.IR	Control	CAM.IR	FLA.IR	SUN.IR
Diet Composition, %												
Wheat	20.0				23.0	23.0	23.0	23.0	27.0	27.0	27.0	27.0
Soybean meal, 46% CP ^2^	39.4				35.7	28.9	30.9	30.4	31.3	25.3	27.3	26.8
Maize	30.04				30.0	29.03	27.03	27.53	29.93	28.95	26.95	27.45
Soybean oil	6.0				7.0				8.0			
Camelina seeds ^3^						15.0				15.0		
Flax seeds ^3^							15.0				15.0	
Sunflower seeds ^3^								15.0				15.0
Dicalcium phosphate	1.83				1.80	1.80	1.80	1.80	1.80	1.80	1.80	1.80
Limestone	1.20				1.00	1.00	1.00	1.00	0.70	0.70	0.70	0.70
NaCl	0.33				0.33	0.33	0.33	0.33	0.33	0.33	0.33	0.33
DL-Met ^4^	0.36				0.33	0.33	0.33	0.33	0.33	0.33	0.33	0.33
L-Lys ^5^	0.34				0.34	0.36	0.36	0.36	0.36	0.34	0.34	0.34
Vitamin-mineral premix ^6^	0.50				0.50	0.25	0.25	0.25	0.25	0.25	0.25	0.25
Sum, %	100				100	100	100	100	100	100	100	100
Chemical composition, g/kg												
ME_n_ (MJ/kg) ^7^	12.45				13.01	12.98	13.02	13.09	13.35	13.20	13.25	13.28
Gross energy (MJ/kg)												
CP ^2^	221.3				209.2	206.2	205.1	207.8	191.5	195.4	195.1	197.8
Lys	14.29				12.75	12.79	12.53	12.82	11.57	11.16	11.75	11.62
Met + Cys	10.51				9.68	9.74	9.31	9.82	8.92	8.79	8.65	9.10
Thr	0.97				0.98	0.97	0.98	0.98	0.73	0.74	0.73	0.75
Trp	0.23				0.12	0.10	0.11	0.13	0.17	0.17	0.16	0.15
Val	1.10				1.00	0.97	0.98	0.99	0.84	0.85	0.83	0.84
Ca	9.84				8.87	8.56	8.28	8.69	7.86	7.86	7.97	8.02
P	6.61				6.29	6.65	6.53	6.37	6.21	6.57	6.59	6.61

^1^ Control, diet without oilseeds; CAM.IR, diet with 15% of infrared-irradiated camelina seeds; SUN.IR, diet with 15% infrared-irradiated sunflower seeds; FLA.IR, diet with 15% infrared-irradiated flax seeds. ^2^ CPl, crude protein; ^3^ IR, infrared irradiation. ^4^ Evonik Degussa Gmbh, Essen, Germany (per kilogram of 990 g methionine) and ^5^ Ajinomoto Eurolysine S.A.S. Amiens, France (per kilogram of 780 g lysine). ^6^ Added minerals and vitamins per kg of starter diet: Mn, 100 mg; I, 1 mg; Fe, 40 mg; Zn, 100 mg; Se, 0,15 mg; Cu, 10 mg; vitamin A, 15,000 IU; vitamin D_3_, 5000 UI; vitamin E, 75 mg; vitamin K_3_, 4 mg; vitamin B_1_, 3 mg; vitamin B_2_, 8 mg; vitamin B_6_, 5 mg; vitamin B_12_, 0.016 mg; biotin, 0.2 mg; folic acid, 2 mg; nicotic acid, 60 mg; pantothenic acid, 18 mg; choline, 1800 mg. Added minerals and vitamins per kg of grower diet: Mn, 100 mg; I, 1 mg; Fe, 40 mg; Zn, 100 mg; Se, 0.15 mg; Cu, 10 mg; vitamin A, 12,000 IU; vitamin D_3_, 5000 UI; vitamin E, 50 mg; vitamin K_3_, 3 mg; vitamin B_1_, 2 mg; vitamin _B2,_ 6 mg; vitamin B_6_, 4 mg; vitamin B_12_, 0.016 mg; biotin, 0,2 mg; folic acid, 1.75 mg; nicotic acid, 60 mg; pantothenic acid, 18 mg; choline, 1600 mg. Added minerals and vitamins per kg of finisher diet: Mn, 100 mg; I, 1 mg; Fe, 40 mg; Zn, 100 mg; Se, 0.15 mg; Cu, 10 mg; vitamin A, 12,000 IU; vitamin D_3_, 5000 UI; vitamin E, 50 mg; vitamin K_3_, 2 mg; vitamin B_1_, 2 mg; vitamin B_2_, 5 mg; vitamin B_6_, 3 mg; vitamin B_12_, 0.011 mg; biotin, 0.05 mg; folic acid, 1.5 mg; nicotic acid, 35 mg; pantothenic acid, 18 mg; choline, 1600 mg. ^7^ ME_n_ = metabolisable energy in the mixtures corrected to zero nitrogen balance.

**Table 3:** Selected productivity parameters and weight of parts of broiler chicken carcasses ^1^.

Items	Treatments ^2^				Statistical Parameters	
	Control	CAM.IR	FLA.IR	SUN.IR	SEM ^3^	*p*-Value ^4^
Slaughter parameters						
Dressing percentage, %	76.6	77.6	77.2	77.8	3.28	0.151
Abdominal fat, % ^5^	0.59 ^a^	0.38 ^b^	0.42 ^b^	0.44 ^b^	0.12	0.016
Muscle weight, % ^5^						
Breast muscle	20.5 ^b,c^	21.39 ^a,b^	23.4 ^a^	18.73 ^c^	2.64	0.019
Thigh muscle	7.79 ^b^	8.71 ^a^	7.91 ^a,b^	8.70 ^a^	1,36	0.022
Drumstick muscle	5.90 ^a,b^	5.77 ^a,b^	6.12 ^a^	5.71 ^b^	0.28	0.031
Organ weight, % ^5^						
Liver	1.84	1.77	1.80	2.12	0.44	0.293
Proventriculus	10.04 ^b^	1.11 ^a,b^	1.14 ^a,b^	1.18 ^a^	0.38	0.027
Heart	0.43	0.45	0.43	0.42	0.01	0.168

^1^ Data represent the mean of 5 cages (10 broiler chickens/cage) per treatment. ^2^ Control, diet without oilseeds; CAM.IR, diet with 15% infrared-irradiated camelina seeds; FLA.IR, diet with 15% infrared-irradiated flax seeds; SUN.IR, diet with 15% infrared-irradiated sunflower seeds; ^3^ SEM, standard error of the mean; ^4^ *p* < 0.05, statistical differences; ^5^ % of final body weight, ^a–c^ statistical differences.

**Table 4:** Content of basic nutrients and mineral elements in natural matter in broiler chicken muscles ^1^.

Items	Treatments ^2^				Statistical Parameters	
	Control	CAM.IR	FLA.IR	SUN.IR	SEM ^3^	*p*-Value ^4^
Breast muscle						
Basic nutrients, g/100 g						
Dry matter	26.1	25.1	23.8	24.7	0.27	0.134
Crude protein ^5^	22.3	23.2	22.9	21.8	0.26	0.127
Ether extract ^6^	1.31 ^a^	1.12 ^b^	1.15 ^b^	1.08 ^b^	0.11	0.031
Crude ash	1.21	1.19	1.18	1.20	0.08	0.153
Energy, kcal	101.0	102.9	102.0	96.9	2.86	0.067
Energy, kJ	422.8	430.7	426.8	405.8	0.45	0.098
Mineral elements, mg/kg						
Ca	28.76 ^b^	32.15 ^a^	34.41 ^a^	28.86 ^a,b^	0.41	0.018
Mg	16.05	17.03	16.16	16.57	0.15	0.153
P	240.1	265.3	247.2	251.4	16.83	0.238
Cu	0.044 ^b^	0.043 ^b^	0.056 ^a^	0.047 ^a,b^	0.03	0.041
Fe	0.475 ^b^	0.503 ^ab^	0.512 ^a^	0.483 ^b^	0.05	0.017
Zn	0.513	0.523	0.517	0.534	0.04	0.238
Drumstick muscle						
Dry matter	26.5	25.3	24.1	24.6	0.11	0.167
Crude protein ^5^	18.3	18.9	17.5	19.4	0.63	0.159
Ether extract ^6^	7.45 ^a^	4.75 ^b^	4.87 ^b,c^	4.49 ^c^	0.05	0.027
Crude ash	1.12	1.09	1.04	1.08	0.07	0.151
Energy, kcal	140.3 ^a^	118.4 ^b^	113.8 ^b^	118.0 ^b^	6.89	0.018
Energy, kJ	587.2 ^a^	495.5 ^b^	476.6 ^b^	494.1 ^b^	0.73	0.021
Mineral elements, mg/kg						
Ca	8.03	8.15	8.21	8.53	0.12	0.267
Mg	22.09	22.18	22.54	22.12	0.53	0.152
P	196.1	218.1	221.4	203.4	4.41	0.171
Cu	0.078	0.096	0.095	0.083	0.05	0.083
Fe	0.641 ^b^	0.686 ^a,b^	0.715 ^a^	0.643 ^b^	0.06	0.024
Zn	1.527	1.521	1.534	1.544	0.05	0.326

^1^ Data represent the mean of 5 cages (2 broiler chickens/cage) per treatment. ^2^ Control, diet without oilseeds; CAM.IR, diet with 15% infrared-irradiated camelina seeds; FLA.IR, diet with 15% infrared-irradiated flax seeds; SUN.IR, diet with 15% infrared-irradiated sunflower seeds; ^3^ SEM, standard error of the mean; ^4^ *p* < 0.05, statistical differences. ^5^ Calculated by Kjeldhal nitrogen N × 6.25. ^6^ Ether extract, crude fat determined following the Soxhlet method, ^a–c^ statistical differences.

**Table 5:** Content of basic nutrients and mineral elements in natural matter in selected broiler chicken organs ^1^.

Items	Treatments ^2^				Statistical Parameters	
	Control	CAM.IR	FLA.IR	SUN.IR	SEM ^3^	*p*-Value ^4^
Liver						
Basic nutrients, g/100 g						
Dry matter	15.9	16.8	17.4	16.8	0.31	0.234
Crude protein ^5^	7.85	8.12	7.69	7.69	0.51	0.087
Ether extract ^6^	1.31 ^a^	1.27 ^a^	1.18 ^b^	1.19 ^b^	0.16	0.035
Crude ash	6.15	6.17	6.16	6.21	0.12	0.147
Energy, kcal	43.19	43.91	41.38	41.47	2.43	0.067
Energy, kJ	180.8	183.8	173.2	173.6	0.65	0.098
Mineral elements, mg/kg						
Ca	9.15 ^c^	10.44 ^b^	11.56 ^a^	9.93 ^b,c^	0.44	0.024
Mg	18.34	17.53	18.09	18.41	0.18	0.208
P	285.8	287.3	284.9	293.5	11.85	0.061
Cu	0.039	0.041	0.045	0.043	0.01	0.075
Fe	9.15	9.45	9.27	9.44	0.06	0.127
Zn	2.67	2.59	2.64	2.61	0.01	0.108
Proventriculus						
Basic nutrients, g/100 g						
Dry matter	29.1	30.4	27.9	28.9	0.06	0.108
Crude protein ^5^	16.8 ^b^	19.1 ^a^	17.2 ^a,b^	18.3 ^a,b^	0.52	0.023
Ether extract ^6^	6.50 ^a^	5.51 ^b^	5.78 ^b^	5.41 ^b^	0.03	0.041
Crude ash	0.891	0.923	1.022	0.934	0.07	0.201
Energy, kcal	125.7	126.0	120.8	121.9	3.2	0.105
Energy, kJ	526.3	527.5	505.8	510.3	0.76	0.123
Mineral elements, mg/kg						
Ca	9.98	9.65	9.51	9.76	0.07	0.141
Mg	12.15	12.74	12.53	12.69	0.38	0.156
P	133.7	141.5	138.4	148.7	2.42	0.203
Cu	0.172 ^b^	0.194 ^a^	0.197 ^a^	0.181 ^a,b^	0.02	0.015
Fe	1.01	1.12	1.16	1.07	0.01	0.138
Zn	1.81	1.87	1.91	1.83	0.05	0.142
Heart						
Basic nutrients, g/100 g						
Dry matter	26.1	25.7	26.9	25.8	0.08	0.118
Crude protein ^5^	15.6	16.3	16.4	15.9	0.48	0.143
Ether extract ^6^	9.50 ^a^	8.60 ^b^	8.16 ^b^	9.61 ^a^	0.05	0.027
Crude ash	0.863	0.901	0.876	0.873	0.07	0.139
Energy, kcal	147.9	142.6	139.04	150.09	7.3	0.252
Energy, kJ	619.2	597.0	582.1	628.4	0.64	0.116
Mineral elements, mg/kg						
Ca	12.01	12.12	12.45	12.78	0.25	0.171
Mg	15.89	16.13	16.09	16.47	0.16	0.262
P	178.2	187.9	184.9	183.1	13.85	0.108
Cu	0.346	0.349	0.351	0.349	0.04	0.235
Fe	4.97 ^b^	6.01 ^a^	6.15 ^a^	6.08 ^a^	0.09	0.021
Zn	6.54	6.87	6.94	6.58	0.12	0.119

^1^ Data represent the mean of 5 cages (2 broiler chickens/cage) per treatment. ^2^ Control, diet without oilseeds; CAM.IR, diet with 15% infrared-irradiated camelina seeds; FLA.IR, diet with 15% infrared-irradiated flax seeds; SUN.IR, diet with 15% infrared-irradiated sunflower seeds; ^3^ SEM, standard error of the mean; ^4^ *p* < 0.05, statistical differences. ^5^ Calculated by Kjeldhal nitrogen N x 6.25. ^6^ Ether extract, crude fat determined following the Soxhlet method, ^a–c^ statistical differences.

**Table 6:** Effect of dietary inclusion of full-fat seeds on haematological indices and content of elements in broiler chicken plasma ^1^.

Items	Treatments ^2^				Statistical Parameters	
	Control	CAM.IR	FLA.IR	SUN.IR	SEM ^3^	*p*-Value ^4^
Haematological indices ^5^						
RBC, 10^12^·L^−1^	2.99	2.86	2.87	2.88	0.05	0.746
HGB, mmol·L^−1^	8.05 ^a^	7.28 ^b^	7.23 ^b^	7.95 ^a^	0.13	0.013
MCHC, mmol·L^−1^	23.97 ^a^	22.49 ^b^	22.49 ^b^	24.45 ^a^	0.27	0.007
MCH, pg	43.57	41.10	40.76	44.43	0.63	0.094
MCV, fl	112.8	113.4	112.4	112.8	0.64	0.965
PCV, l·L^−1^	0.34	0.32	0.32	0.32	<0.01	0.604
Plasma elements						
Ca, mmol·L^−1^	2.42 ^a^	2.19 ^b,c^	2.35 ^a,b^	2.09 ^c^	0.04	0.023
Mg, mmol·L^−1^	0.86	0.80	0.80	0.75	0.02	0.106
P, mmol·L^−1^	1.91	1.86	2.05	1.80	0.05	0.277
Cu, µmol·L^−1^	6.02	6.36	4.98	5.37	0.27	0.125
Fe, µmol·L^−1^	14.04 ^b^	14.88 ^a,b^	17.80 ^a^	17.77 ^a^	0.58	0.003
Zn, µmol·L^−1^	22.42	21.37	23.15	23.47	0.83	0.103

^1^ Data represent the mean of 5 cages (2 broiler chickens/cage) per treatment. ^2^ Control, diet without oilseeds; CAM.IR, diet with 15% camelina seeds; FLA.IR, diet with 15% flax seeds; SUN.IR, diet with 15% sunflower seeds; ^3^ SEM, standard error of the mean; ^4^ *p* < 0.05, statistical differences; ^5^ RBC, red blood cell; HGB, haemoglobin; MCHC, mean corpuscular haemoglobin concentration; MCH, mean corpuscular haemoglobin; MCV, mean corpuscular volume; PCV, packed cell volume. ^a–c^ statistical differences.

## Data Availability

Data available upon request.
